# Selection of candidate genes controlling veraison time in grapevine through integration of meta-QTL and transcriptomic data

**DOI:** 10.1186/s12864-019-6124-0

**Published:** 2019-10-15

**Authors:** Pietro Delfino, Sara Zenoni, Zahra Imanifard, Giovanni Battista Tornielli, Diana Bellin

**Affiliations:** 1 Department of Biotechnology, University of Verona, Strada le Grazie 15, 37134 Verona, Italy; 2 Present address: Department of Diagnostics and Public Health, Section of Pathology, University and Hospital Trust of Verona, Verona, Italy

**Keywords:** Grapevine, Phenology, QTL, Meta-QTL, Transcriptomics, Veraison, Grape berry development

## Abstract

**Background:**

High temperature during grape berry ripening impairs the quality of fruits and wines. Veraison time, which marks ripening onset, is a key factor for determining climatic conditions during berry ripening. Understanding its genetic control is crucial to successfully breed varieties more adapted to a changing climate. Quantitative trait loci (QTL) studies attempting to elucidate the genetic determinism of developmental stages in grapevine have identified wide genomic regions. Broad scale transcriptomic studies, by identifying sets of genes modulated during berry development and ripening, also highlighted a huge number of putative candidates.

**Results:**

With the final aim of providing an overview about available information on the genetic control of grapevine veraison time, and prioritizing candidates, we applied a meta-QTL analysis for grapevine phenology-related traits and checked for co-localization of transcriptomic candidates. A consensus genetic map including 3130 markers anchored to the grapevine genome assembly was compiled starting from 39 genetic maps. Two thousand ninety-three QTLs from 47 QTL studies were projected onto the consensus map, providing a comprehensive overview about distribution of available QTLs and revealing extensive co-localization especially across phenology related traits. From 141 phenology related QTLs we generated 4 veraison meta-QTLs located on linkage group (LG) 1 and 2, and 13 additional meta-QTLs connected to the veraison time genetic control, among which the most relevant were located on LG 14, 16 and 18. Functional candidates in these intervals were inspected. Lastly, taking advantage of available transcriptomic datasets, expression data along berry development were integrated, in order to pinpoint among positional candidates, those differentially expressed across the veraison transition.

**Conclusion:**

Integration of meta-QTLs analysis on available phenology related QTLs and data from transcriptomic dataset allowed to strongly reduce the number of candidate genes for the genetic control of the veraison transition, prioritizing a list of 272 genes, among which 78 involved in regulation of gene expression, signal transduction or development.

## Background

Grapevine is one of the most important fruit crops grown worldwide. It provides berries to be used as fresh fruit, raisins or for wine making, a key socio-economic sector for many countries.

Grapevine’s developmental cycle is described by three main phenological stages. Budbreak represents the onset of the vegetative growth. Flowering starts the vine reproductive growth, leading, when fertilisation takes place, to berry formation. Veraison, the onset of the berry ripening process, is the stage when major changes occur in berries. Indeed, while organic acids and a few other compounds already accumulate in berries before this stage, starting from veraison time berries switch from being small, hard and acidic to a status where they become larger, softer, coloured and accumulate sugars and flavours or aromatic compounds with a decrease in organic acids content. All these events largely determine wine quality [1]. Finally, when berries meet required sugar and acidity content they are harvested, even though maturity, also called ripening, cannot be considered as true a phenological stage, due to the difficulties in establishing uniform criteria for different varieties [2].

Grapevine phenology is driven by temperature and, being also under genetic control, different varieties differ in their phenological timing, due to morphological and physiological characteristics [3]. Accordingly, the suitability of each variety to a given area has been defined by climatic factors that limit their geographic distribution, with the finest wines associated with geographically distinct viticulture regions [4]. Veraison date determines climatic conditions during the ripening. Too high temperatures lead to negative effects, which include the reduction of final anthocyanin content in berries [5], with consequences on the visual aspect of the fruit and red wines, the decoupling of ripening parameters (i.e., excess sugar content but low acidity, [6, 7]), an inadequate pool of polyphenolic compounds and incomplete development of flavours. Long-term studies on climatology and grapevine phenology demonstrated that global warming has already affected, in several areas, the onset and duration of phenological events, with an acceleration in their timing [4, 8–14]. Further changes and their impacts on quality have been also modelled either globally or for the most important grape growing areas worldwide, highlighting that impacts of climate change on viticulture suitability is expected to become substantial, at least for some regions [2, 15–19]. Adaptation by taking into account agronomic practices or migration of vineyards is unlikely, and incorporation through cross-breeding of traits for the control of phenology beside temperature resilience is recommended in the long term [2, 15, 20, 21].

With the final aim of breeding varieties better adapted to future climatic conditions many teams have attempted to elucidate the genetic determinism of phenology, and in particular veraison time, by applying QTL studies, finding regions in the grapevine genome linked to observed variation including a large number of genes [22–26]. An interesting opportunity to summarize available QTL information and refine their genomic location, by comparing individual experiments narrowing down original intervals, comes from QTL meta-analysis [27, 28]. Indeed, QTLs detected in different experiments and located in a given region of a chromosome could possibly represent several estimations of one single shared QTL. This hypothesis can be tested by appropriate statistical tools that indicate the most likely number of ‘real’ QTLs underlying co-located QTLs. The resulting meta-QTLs are expected to better refine the boundaries of the causative genomic intervals by integrating information from different studies. This approach was first applied to study flowering time in maize [29]. Subsequent positional cloning and association mapping analysis revealed two genes in meta-QTL intervals effectively involved in modulating flowering time [30–32] confirming meta-analysis as useful tool for predicting candidates and developing markers for breeding. Since then QTL meta-analyses have become popular in the literature to score QTLs of huge breeding potential and towards QTL validation and/or prioritization of candidates. Lately, meta-analysis has been successfully applied in many crops like rice [33], cotton [34], potato [35], soybean [36], bean [37] and many others. However, this approach has so far not been applied in grapevine.

Technological advances and the availability of a high-quality draft of the grapevine genome sequence [38] have encouraged characterizations of berry development at the transcriptomic level [39–48]. Beside identifying specific transcripts modulated during berry development, these studies revealed that a major transcriptomic shift is associated to the veraison transition [44, 49]. Integrated network analysis of expression data allowed genes to be classified according to their correlation with interaction partners, and to define “switch genes,” likely playing a key role in this major transition [44, 46]. Lately, by detailed analysis of expression profiles in two different varieties, two rapid and successive transitions at the timing of the molecular reprogramming associated to veraison were defined, including positive and negative molecular “biomarkers” [48].

The number of candidate genes putatively involved in the genetic control of the veraison transition either underlying veraison QTLs, or emerging from transcriptomic studies, is huge. With the final aim of defining a prioritizing strategy we developed a consensus genetic map from 39 independent maps and, following QTLs projection, performed a meta-analysis of co-located veraison QTLs or of veraison QTLs and other phenology QTLs. Then, by anchoring to the grapevine genome assembly and integrating information from transcriptomic studies, we selected a set of putative key regulators for the grapevine veraison transition.

## Results

### Selection of grape QTLs for integration and scoring of phenology related ones

All published grapevine QTL studies up to October 2018 were collected from the literature to retrieve those including suitable information for integration of data from independent studies/populations. This resulted in the selection of 42 publications, reporting 47 different QTL maps from more than 80 available (Additional file 1) [22–26, 50–86]. These QTL studies exploited a total of 24 different cross populations, constituted on average by 157 offspring (number of offspring ranging from 74 to 265). Cross populations were mainly F1, with the only exception of two populations obtained by self-pollination and one obtained by selfing an F1 [56, 67, 68]. A large number of cross populations (14) were derived by crossing *Vitis vinifera* with hybrids or other *Vitis* species, but a number of intra-vinifera crosses was also reported.

These selected QTL studies included 2093 QTLs for 354 different phenotypes. A detailed list of all scored phenotypes, grouped according to the study and including the QTL short name used in the relative reference as well as a short description, is provided in Additional file 2. Each measured phenotype/QTL was manually attributed to its most related trait, for which the phenotype was considered to be a descriptor, and traits were arbitrarily grouped in 8 main trait categories. An overview of the plant traits in grapevine currently more characterized by these studies, grouped according to the eight different trait categories, is given in Additional file 3. Number of QTLs for each trait as well as number of studies considering each trait are shown. The trait for which the highest number of QTLs is currently available in the literature is berry metabolites (Additional file 3a). This is expected since high throughput metabolomic approaches can easily produce a large amount of data. However, the overall most scored trait across QTL studies was berry weight (scored in 12 independent studies), while trait categories most addressed by independent studies so far have been phenology and pathogen resistance (Additional file 3b, number of independent studies per category indicated in brackets).

Interestingly, 184 QTLs among those included in the selected studies were belonging to the trait category phenology, derived from 12 publications. This category includes 5 main traits namely bud burst, flowering, veraison, ripening times and length of intervals among the different stages. Table 1 provides details about these phenology QTLs including the reference in which they were found, the short name attributed to the originally scored phenotype in each of these publications and the distribution across the different traits’ type. Among these, 54 QTLs derived from 6 studies were related to the main trait veraison.

**Table 1:** Published grapevine phenology related QTLs included in the analysis

QTL study reference	Trait	QTL Short_Name^a^	N° of QTLs included in the study
[52] Ban et al. 2016	ripening	ta, ssc	5
[53] Bayo Canha, PhD thesis 2015	bud break	Sp	6
	flowering	Fw	3
	interval	Vr-Rp	1
	ripening	Tss/Ac, Ac, Ma, Tar/Ma, Rp	11
	**veraison**	**Vr**	**2**
[58] Carreño Ruiz, PhD thesis 2012	bud break	BB	2
	flowering	FT	7
	ripening	RT	4
	**veraison**	**VT**	**6**
[22] Costantini et al. 2008	flowering	FT	4
	interval	F-R, F-V, V-R	6
	ripening	R	1
	**veraison**	**VP, VT**	**4**
[23] Duchêne et al. 2012	bud break	B-B	5
	interval	B-F, F-V	17
[25] Fechter et al. 2014	flowering	FBL, FS	27
	**veraison**	**VT**	**4**
[24] Grzeskowiak et al. 2013	bud break	BB	1
	flowering	FB	3
	**veraison**	**VB, VE**	**20**
[75] Mejía et al. 2007	ripening	RDA	3
[83] Viana et al. 2013	ripening	At, Bpc	9
[86] Zhao et al. 2016	ripening	Cma	5
[85] Zhao et al. 2015	ripening	SSC	2
[26] Zyprian et al. 2016	interval	F-V	8
	**veraison**	**VT**	**18**

^a^The original short name of phenology QTLs included in the analysis is indicated. QTLs for veraison are highlighted in bold. QTL short name abbreviations are as following: *ta* total acid, *ssc* solubile solids concentration, *Sp* Sprouting, *Fw* Flowering, *Vr-Rp* Veraison-ripening period, *Tss/Ac* Ratio of total soluble solids to total acidity, *Ac* Total acidity, *Ma* Malic acid, *Tar/Ma* Ratio of tartaric acid to malic acid, *Rp* Ripening, *Vr* Veraison, *BB* Bud break, *FT* Flowering time, *RT* Ripening time, *VT* Veraison time, *F-R* Flowering ripening interval, *F-V* Flowering-veraison interval, *V-R* Veraison ripening interval, *R* Ripening, *VP* Veraison period, *B-B* February–budbreak/Bud, *B-F* Budbreak–flowering/Flo, *FBL* Time of full bloom, *FS* Start of flowering, *FB* Flowering beginning, *VB* Veraison beginning, *VE* Veraison end, *RDA* Ripening date, *At* Tartaric acid, *Bpc* Brix per cluster, *Cma* Fruit maturation period. Further details about phenotype scoring for each QTL can be found in the Additional file 2, Additional file 19 or in the indicated original publications.

### Building of a grapevine consensus genetic map

All 35 genetic maps used in the selected QTL studies (see genetic map references in Additional file 1) were used as input for the construction of a consensus map in BioMercator v4.2 software [87]. A grapevine reference map, developed from the integration of 5 different genetic maps [88], was also included, as well as 3 further available grapevine genetic maps [22, 89, 90].

Common markers made the construction of a consensus possible for each of the 19 grapevine chromosomes with no residual conflicts (Additional files 4 and 5). The consensus map consisted of 19 linkage groups, corresponding to the 19 grapevine chromosomes, including 3130 markers with a total length of 1922 cM and an average number of markers and length per linkage group of 164 and 101 centiMorgan (cM) respectively. The number of markers shared by at least two maps was 1209, corresponding to 38.63% of the total markers, with an average of 63 shared markers per linkage group (Table 2). The number of maps used for the construction of each linkage group varied from 26 (LG 11) to 39 (LGs 1, 2, 4, 5, 10, 12, 17, 18), due to the different number of markers shared among maps (Table 2, Additional file 6).

**Table 2:** Consensus genetic map features

LG	N° of markers	N° unique markers^a^	N° markers in at least two maps	Length (cM)	N° of individual maps integrated
I	214	130	84	95.68	39
II	130	71	59	89.73	39
III	135	92	43	92.03	37
IV	161	101	60	93.36	39
V	206	150	56	70.64	39
VI	139	93	46	90.72	38
VII	204	124	80	82.09	38
VIII	167	88	79	95.72	37
IX	128	77	51	85.01	35
X	168	93	75	141.87	39
XI	90	38	52	72.64	26
XII	211	153	58	143.13	39
XIII	156	89	67	113	37
XIV	202	118	84	94.79	37
XV	128	86	42	93.44	37
XVI	126	74	52	68.7	35
XVII	130	76	54	104.47	39
XVIII	275	172	103	148.93	39
XIX	160	96	64	146.03	37
Total	3130	1921	1209	1921.98	

^a^Markers present in only one input genetic map

Marker density was not equally distributed among the consensus, with peaks in putative centromeric positions similarly to those found in original maps. However, comparison of markers order between the single component maps and consensus map revealed a high level of correlation (Additional file 7). Spearman’s rank correlation values of pairwise comparisons were significantly high for all maps but two, possibly due to the low number of shared markers.

The consensus genetic map was connected to the genome annotation through the use of an anchor file including marker’s physical position, recovered as explained in the methods section. Upon removal of markers showing incongruent or not unique physical positions 713 markers (on average 38 markers per LG) were physically mapped on the 12X.v2 assembly of the grapevine genome [91]. Their physical coordinates are also included in the map file Additional file 4. Among these markers, 480 (67%) were shared by at least two original maps, and the majority (513, 72%) were microsatellite markers.

### Distribution of grapevine QTLs on the consensus genetic map

All QTLs from the 47 selected QTL studies (Additional file 1) were projected onto the consensus map to build QTL consensus maps for each trait (Additional file 8). In total 1899 QTLs (91%) could be successfully plotted while 194 could not be projected, due to lack of anchoring markers. The percentage of plotted QTLs was comparable across the different traits, ranging from 82 to 100%. Only for the traits ripening time, fertility and black rot resistance the number of plotted QTLs was lower (75, 65 and 65% respectively).

**Fig. 1:**
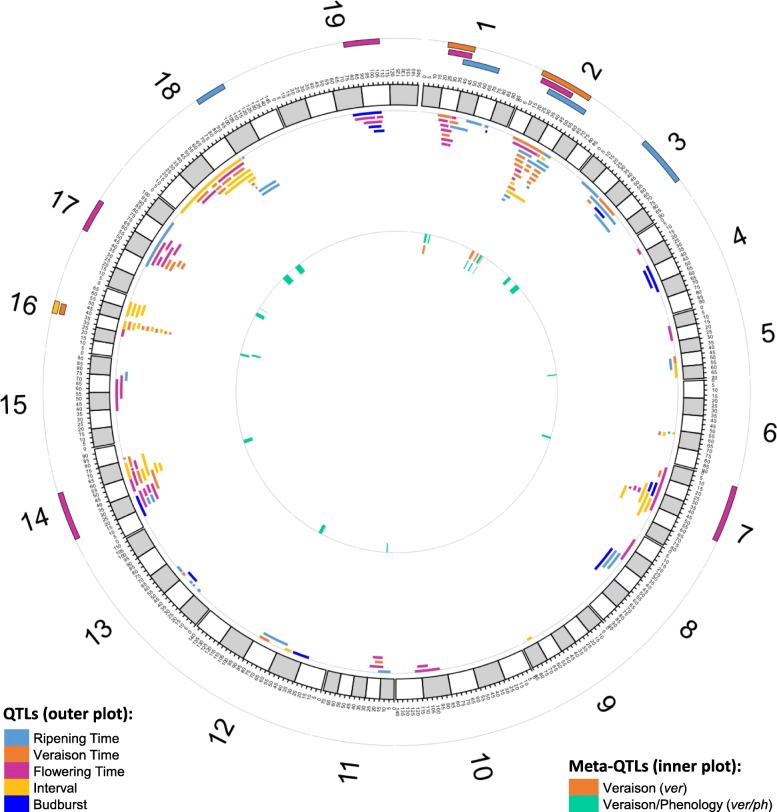
Phenology consensus QTL map with meta-QTLs positions. Consensus QTL map for phenology related traits built by plotting of original QTLs on the consensus map (outer plot). QTLs positions are indicated on the internal side of each chromosome. Genetic regions spanned by QTLs confirmed in independent populations are highlighted by a bar on the outer side of each chromosome. Meta-QTLs (inner plot) were calculated from overlapping veraison QTLs and from veraison QTL overlapping to other phenology QTLs. Colour code for each trait is given in the legend tables

To aid spotting QTLs emerging independently in more studies, circular plots of consensus QTL maps were prepared for each trait grouped by trait category. Plots for all trait categories except phenology are provided in Additional file 9, while the phenology category plot is provided in Fig. 1. Careful inspection revealed that the trait with the highest number of co-located QTLs from independent studies, highlighted by bars on the outer side of chromosomes, was downy mildew resistance (Additional file 9e). QTLs located on LG 1, 4, 5, 6, 7, 8, 10, 12, 14, 17 and 18 were all confirmed by more studies, with up to 5 studies mapping QTLs to an overlapping region on LG 18. However, for all other pathogen resistance traits only one QTL was discovered by different studies, namely the QTL on LG 15 for powdery mildew resistance found in three independent studies. In a similar way, several QTLs for the trait anthocyanin, on LG 1, 2, 4, 6, 7, 10, 12 and 17, were all confirmed by independent studies with the most consistently found QTL mapping on LG 2. However, also for this category no other trait revealed confirmed QTLs, at least considering these studies, since the overlapping QTLs shown in Additional file 9d for other traits in this category all come from one study. For abiotic stress and cluster related traits categories, overlaps among QTLs from independent studies were also few and involved just one trait inside the category (Additional file 9a and b). Instead, for the categories berry morphology, seeds related traits and vegetative traits more than one traits had overlapping QTLs mapped in independent studies (Additional file 9c, f and g). Importantly phenology was overall the category for which the number of QTLs’ kind confirmed by independent studies was the highest. Indeed, in this category four ripening QTLs were independently found at similar genetic loci by independent studies, i.e. on LG 1, 2, 3, 18, six flowering QTLs were consistently mapped to LG 1, 2, 7, 14, 17 and 19, while for veraison and flowering-veraison interval 3 and 1 confirmed QTLs were mapped respectively on LG 1, 2 and 16 or on LG 16 (Fig. 1). However, for each of these QTLs the confirmation was only based on few studies, ranging from 2 to a maximum of 3 (Additional file 10).

### Narrowing down of candidates for veraison time by meta-QTL analyses

For the purpose of performing a meta-analysis on veraison co-located QTLs independently mapped by more studies, thus reducing their genetic intervals, the list of veraison QTLs projected onto the consensus map (Additional file 8) was manually curated, as explained in the material and methods section, to avoid over-representation of any trait. We selected 35 veraison QTLs from 6 studies [22, 24–26, 53, 58]. Meta-analysis was applied to LG 1 and 2, where overlapping QTLs were retained after pruning. Our meta-analysis resulted in the identification of 4 veraison meta-QTLs (*ver*), one located on LG 1 (*ver_1.1*) and three on LG 2 (*ver_2.1, ver_2.2* and *ver_2.3*) (Table 3, Fig. 1). More in detail, the veraison meta-QTL on LG 1 was derived from 2 original co-located QTLs while on LG 2 each resulted from the integration of 5 to 7 original co-located QTLs. Average confidence interval (CI) was 3.5 cM ranging from 1.2 cM for *ver_2.3* to 5.1 cM for *ver_2.1*, which was the largest one. On LG 1 the original CI covered by QTLs was reduced from 23.9 cM to 4.3 cM (5.6 times) by the meta-analysis. On LG 2 the reduction of CI was 5 times overall, with a strongest effect on the *ver_2.3* meta-QTL. *R*^*2*^ values of meta-QTLs were all higher than 10%. In particular, *ver_2.2* was the most relevant, explaining up to 34% of total variance.

**Table 3:** Meta-QTLs calculated from overlapping veraison QTLs

LG	Meta-QTL	Peak Pos. (cM)	Mean R^2^	Start (cM)	End (cM)	Start (bp)^a^	End (bp)^a^	Pos Candidates^a^	Original QTLs co-located^b^	QTL Studies (Populations)^b^	QTL^c^	Ref^d^
I	***ver_1.1***	31.29	0.11	29.15	33.43	2,510,506	3,254,952	78	2	2	**VT**	[25, 26]
II	***ver_2.1***	31.34	0.17	28.79	33.89	4,029,921	5,344,816	147	7	2	**VB, Vr**	[24, 53]
	***ver_2.2***	41.55	0.13	40.00	43.30	5,717,649	7,154,894	96	5	3	**Vr, VB, VE, VT**	[22, 24, 53]
	***ver_2.3***	53.47	0.34	52.88	54.07	13,336,750	16,677,137	94	5	3	**Vr, VE, VP**	[22, 24, 53]

^a^Physical position and number of positional candidates were derived by anchoring of the genetic map to grape genome assembly 12X.v2

^b^Both number of original QTLs for the meta-QTL calculation as well as the number of independent populations from which the overlapping QTLs were found are given.

^c^The kind of overlapping veraison QTLs used for the analysis is indicated. Veraison QTLs are highlighted in bold. QTL short name abbreviations are as following: *Vr* Veraison, *VT* Veraison time, *VP* Veraison period, *VB* Veraison beginning, *VE* Veraison end. Further details about phenotypes scoring can be found in the Additional file 2, Additional file 19 or in the indicated original publications.

^d^Reference of the study in which the QTLs included in the meta-QTL analysis were found

Inspection of the consensus QTL map for the whole phenology category (Fig. 1) revealed extensive co-localization also across the different traits (i.e. co-location of veraison and ripening QTLs, etc.). Co-location of veraison QTLs with other phenology QTLs was indeed highly significant compared to a random distribution (*χ*^2^-test, *p < 0.01*) [92]. Overlapping phenology QTLs could represent several estimates of a single QTL affecting more developmental stages, which would justify the attempt to identify consensus QTLs across different phenology traits. In agreement with such an option, a meta-analysis for veraison QTLs and overlapping QTLs for other phenology traits was applied by considering 141 phenology QTLs retained after plotting and pruning of those in the consensus map (Additional file 8), similarly to what was previously described. When applied to veraison QTLs mapping on LG 1 and LG 2, this approach identified meta-QTLs largely overlapping with previously reported veraison meta-QTLs (Additional file 11). Therefore, with the final aim of reducing the number of genes underlying veraison QTLs, the same strategy was applied to veraison QTLs on other LGs, and this identified 13 further indicative meta-QTL regions potentially relevant for the genetic control of veraison (Table 4, Fig. 1). We named these additional meta-QTLs as *ver/ph* to clarify that they were obtained from veraison QTLs overlapping with other phenology related QTLs. Among these, two meta-QTLs on LG 16 were particularly relevant, explaining up to 35 and 38% of total phenotypic variance.

**Table 4:** Meta-QTLs calculated from veraison QTLs overlapping with other phenology QTLs

LG	Meta-QTL	Peak Pos. (cM)	Mean R^2^	Start (cM)	End (cM)	Start (bp)^a^	End (bp)^a^	Pos Candidates^a^	Original QTLs co-located^b^	QTL Studies (Populations)^b^	QTL^c^	Ref.^d^
III	***ver/ph_3.1***	27.67	0.15	24.43	30.92	560,404	1,647,064	138	5	3	**VT**, SSC, Bpc	[58, 83, 85]
	***ver/ph_3.2***	50.42	0.14	45.30	55.54	5,903,464	10,894,193	288	4	3	**V**T, SSC, Bpc, BB	[58, 83, 85]
V	***ver/ph_5.1***	50.97	0.09	49.77	52.18	16,799,689	19,536,797	111	3	2	**VT**,F-V, Ma	[26, 53]
VII	***ver/ph_7.1***	9.59	0.16	7.58	11.60	1,087,707	1,552,842	59	2	2	**VT**, Fw	[53, 58]
XI	***ver/ph_11.1***	16.15	0.11	15.01	17.30	2,934,932	3,356,851	50	4	2	FBL, FS, Tar/Ma, **VT**	[25, 53]
XII	***ver/ph_12.1***	77.85	0.19	74.31	81.40	23,793,458	24,155,112	27	2	2	**VT**, RT	[26, 58]
XIV	***ver/ph_14.1***	55.03	0.22	51.45	58.62	22,441,297	24,645,689	157	7	4	B-F, FS, FT, **VT**	[23, 25, 26, 58]
XVI	***ver/ph_16.1***	34.70	0.31	32.53	36.88	14,012,548	16,583,139	126	4	2	F-V, **VT**	[22, 26]
	***ver/ph_16.2***	38.49	0.38	36.49	40.50	16,503,904	17,318,604	51	5	2	F-V, **VT**	[23, 26]
XVII	***ver/ph_17.1***	48.83	0.13	45.12	52.54	4,969,509	6,401,642	113	6	3	FBL, FS, RDA, **VB**	[24, 25, 75]
	***ver/ph_17.2***	61.83	0.11	61.46	62.20	8,920,888	9,063,993	12	7	4	FBL, FS, RDA, **VB**, F-V	[24–26, 75]
XVIII	***ver/ph_18.1***	34.68	0.17	28.21	41.15	1,836,848	5,349,350	322	2	2	**VT**, FT	[26, 58]
	***ver/ph_18.2***	66.33	0.13	60.57	72.10	10,927,035	15,526,564	330	4	3	**VT**, FT, F-V	[23, 26, 58]

^a^Physical position and number of positional candidates were derived by anchoring of the genetic map to grape genome assembly 12X.v2

^b^Both number of original QTLs for the meta-QTL calculation as well as the number of independent populations from which the overlapping QTLs were found are given.

^c^The kind of overlapping phenology related QTLs used for the analysis is indicated. Veraison QTLs overlapping with other phenology QTLs are highlighted in bold. QTL short name abbreviations are as following: *ssc* solubile solids concentration, *Fw* Flowering, *Ma* Malic acid, *Tar/Ma* Ratio of tartaric acid to malic acid, *BB* Bud break, *FT* Flowering time, *RT* Ripening time, *VT* veraison time, *F-V* Flowering-veraison interval, *B-F* Budbreak–flowering/Flo, *FBL* Time of full bloom, *FS* Start of flowering, *VB* veraison beginning, *RDA* Ripening date, *Bpc* Brix per cluster. Further details about phenotypes scoring can be found in the Additional file 2, Additional file 19 or in the indicated original publications.

^d^Reference of the study in which the QTLs included in the meta-QTL analysis were found

**Fig. 2:**
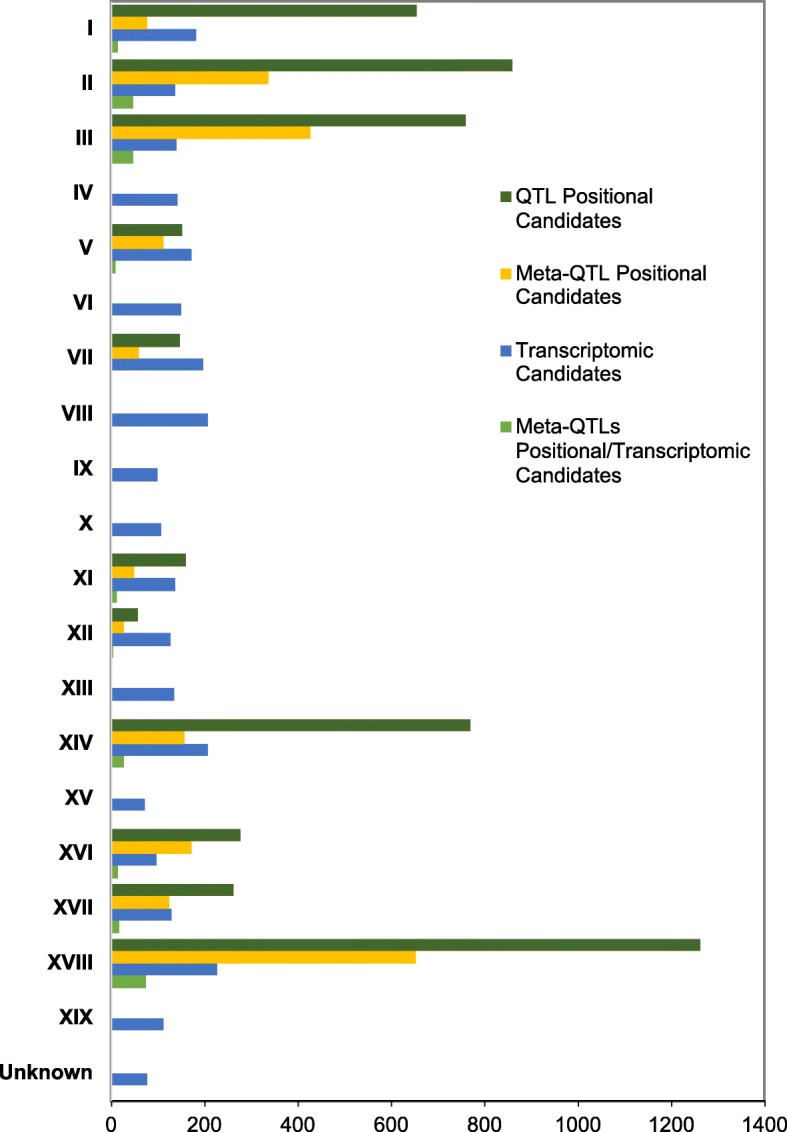
Reduction in number of candidate genes for the genetic control of veraison time by the integrated approach. Number of candidate genes for veraison time control in each chromosome selected by QTLs studies, meta-QTL analysis, transcriptomic analysis or by the integrated approach is shown

In conclusion, after anchoring to the genome, the number of genes underlying original veraison QTLs was narrowed down by applying the meta-QTL analysis, by a factor of 3.7. By including alternative phenology related traits, we also reduced the number of positional candidates at further locations, even though to a lesser extent (2.2 times) (Fig. 2). However, we should consider that this last approach relies on the assumption of shared genetic control, which could lead to skipping relevant candidates, if not verified. Lists of candidate genes in *ver* meta-QTLs and *ver/ph* meta-QTLs intervals, with the corresponding CRIBIv1 annotation (http://genomes.cribi.unipd.it/gb2/gbrowse/public/vitis_vinifera_v2/), are given in Additional files 12 and 13 respectively.

To validate our meta-QTL procedure a similar analysis was applied to QTLs projected on the consensus for the anthocyanins trait. We focused on overlapping QTLs mapping on LG 2. Indeed, berry colour genetic control has already been elucidated and linked to two adjacent regulatory genes, the *VvMYBA1* and *VvMYBA2* genes, located on chromosome (Chr) 2 [93, 94]. The meta-QTL analysis on 28 overlapping QTLs derived from 5 independent studies identified 7 meta-QTLs (Additional file 14). The *VvMYBA1* and *VvMYBA2* genes were both included in the list of the 125 genes underlying these meta-QTLs (Additional file 15).

### Selection of meta-QTL candidate genes differentially expressed across veraison

As further alternative to reduce the number of candidates and prioritize them, we integrated positional information derived from the meta-QTL approach with molecular information obtained in previous transcriptomic studies.

Firstly, the 2195 positional candidates underlying veraison meta-QTLs *ver* or *ver/ph* (Additional files 12 and 13) were explored for their expression profiles in different organs, according to the grapevine expression atlas [49]. Four hundred and thirteen genes were never expressed either in berry, rachis or seed and were thus excluded.

Transcriptomic changes in berries during development and in particular across veraison have been widely explored. Taking advantage of previous transcriptomic studies we constituted a list of molecular candidates putatively involved in the veraison time control and compared them with our remaining positional candidates. From a transcriptomic dataset including berries collected at four time points of development (pea-size, beginning to touch, softening and full ripe), in 10 different grapevine genotypes, a massive transcriptomic change was found to be associated to the veraison transition, and 1478 genes commonly differentially expressed across veraison in all genotypes, were identified [44, 46]. Moreover, a recent transcriptomic map of berry development analysing weekly gene expression in Pinot Noir over 3 years allowed to define two rapid and successive transitions at the timing of the molecular reprogramming of berry development associated to veraison and identified positive and negative molecular “biomarkers” of these transitions [48]. This RNA-Seq dataset was further inspected in this study, especially at early time points before veraison, searching for first molecular events associated to veraison by looking for the transition across which the highest number of such “biomarkers” was differentially expressed in each of the 3 years (Additional file 16). This transition represents an early stage before veraison when the transcriptomic rearrangement associated to veraison starts to occur. One thousand seven hundred forty-nine genes mainly modulated in their profiles across this transition in at least two of the 3 years were then selected. By combining these genes with the 1478 genes identified in the 10 genotypes a final list of 2850 genes, representing transcriptomic candidates for veraison time control, was created (Additional file 17).

We found that among the 1782 positional candidates located under meta-QTLs and expressed in berry, rachis or seed, 272 genes are also transcriptomic candidates (Additional file 18). In detail, 61 lays under *ver* meta-QTLs, and include 16 genes encoding for proteins involved in regulation of gene expression, signalling or development (Table 5). Beside these, other genes belonging to functional classes like transport (7 genes) or carbohydrate metabolism (5 genes), like, for example, a vacuolar invertase, were found, which could also be potentially involved in the genetic control of veraison time mapped at these locations. The other 211 genes co-localized instead to *ver/ph* meta-QTLs. Among these, 62 were involved in regulation of gene expression, signal transduction or development according to their gene ontologies (GO) annotation (Table 5). Moreover, representatives of other relevant functional classes, mainly enzymes involved in carbohydrate metabolism or transporters for sugar related compounds, were also among candidates found at these locations.

**Table 5:** Transcriptomic candidates underlying *ver* meta-QTLs or *ver/ph* meta-QTLs selected by GO annotation for gene expression regulation, signalling or development

Gene ID^a^	Chr	Start	End	Annotation	mQTL^b^	Transcriptomic candidate^c^
VIT_01s0011g02950	1	2,618,690	2,632,669	Zinc finger (C3HC4-type ring finger)	*ver_1.1*	[48]
VIT_01s0011g03070	1	2,751,566	2,753,036	ERF/AP2 Gene Family (VvRAV1)	*ver_1.1*	[44, 46]
VIT_01s0011g03520	1	3,190,826	3,192,777	Constans-like 16	*ver_1.1*	[44, 46]
VIT_02s0025g04730	2	4,100,066	4,103,095	Glyoxylate reductase	*ver_2.1*	[44, 46]
VIT_02s0154g00070	2	4,804,832	4,807,460	Abnormal floral organs	*ver_2.1*	[44, 46]
VIT_02s0154g00080	2	4,813,347	4,818,031	Multi-copper oxidase (SKU5)	*ver_2.1*	[44, 46]
VIT_02s0012g00310	2	6,204,735	6,223,899	Lon protease	*ver_2.2*	[48]
VIT_02s0012g00570	2	6,554,241	6,560,259	Pseudo-response regulator 2 (APRR2) (TOC2)	*ver_2.2*	[44, 46]
VIT_02s0012g00590	2	6,600,611	6,610,281	Unknown protein	*ver_2.2*	[48]
VIT_02s0012g00990	2	7,043,508	7,046,965	LOL1 (LSD ONE like 1)	*ver_2.2*	[44, 46]
VIT_02s0012g01010	2	7,087,110	7,089,452	Leucine-rich repeat	*ver_2.2*	[48]
VIT_02s0012g01040	2	7,120,118	7,122,681	NAC domain-containing protein (VvNAC13)	*ver_2.2*	[44, 46, 48]
VIT_02s0033g00300	2	14,144,838	14,148,929	myb family	*ver_2.3*	[44, 46]
VIT_02s0033g00390	2	14,291,727	14,292,732	VvMybA2	*ver_2.3*	[44, 46]
VIT_02s0033g00410	2	14,351,791	14,352,807	VvMybA1	*ver_2.3*	[44, 46]
VIT_02s0033g00450	2	14,420,525	14,421,283	VvMybA3	*ver_2.3*	[44, 46, 48]
VIT_03s0038g00860	3	689,247	693,308	Basic Leucine Zipper Transcription Factor (VvbZIP05)	*ver/ph_3.1*	[48]
VIT_03s0038g01090	3	862,995	863,398	Auxin responsive SAUR protein	*ver/ph_3.1*	[44, 46]
VIT_03s0038g01110	3	866,357	866,897	Auxin-responsive SAUR31	*ver/ph_3.1*	[44, 46]
VIT_03s0038g01310	3	921,733	927,965	Auxin responsive SAUR protein	*ver/ph_3.1*	[48]
VIT_03s0038g02130	3	1,468,239	1,469,371	Cold shock protein-1	*ver/ph_3.1*	[48]
VIT_03s0180g00040	3	5,973,785	5,975,813	Cyclin D3_2	*ver/ph_3.2*	[48]
VIT_03s0091g00210	3	6,507,392	6,509,263	Ethylene-responsive protein	*ver/ph_3.2*	[44, 46]
VIT_03s0091g00260	3	6,548,677	6,549,577	Zinc finger protein 4	*ver/ph_3.2*	[44, 46, 48]
VIT_03s0091g00870	3	7,342,165	7,357,074	Adenylylsulfate kinase 1 (AKN1)	*ver/ph_3.2*	[48]
VIT_03s0091g01060	3	7,673,917	7,675,754	Cyclin delta-2	*ver/ph_3.2*	[48]
VIT_03s0088g00290	3	8,315,170	8,315,924	Phytosulfokines PSK2	*ver/ph_3.2*	[44, 46]
VIT_03s0088g01180	3	9,438,885	9,442,060	Proline iminopeptidase	*ver/ph_3.2*	[48]
VIT_05s0062g00760	5	19,469,712	19,473,848	Receptor kinase RHG4	*ver/ph_5.1*	[48]
VIT_11s0016g03640	11	2,972,017	2,974,625	Rac-like GTP-binding protein ARAC7 (GTPase protein ROP9)	*ver/ph_11.1*	[44, 46]
VIT_11s0016g03650	11	2,976,690	2,979,682	CDKF;1 (CDK-activating kinase 1A	*ver/ph_11.1*	[48]
VIT_11s0016g03880	11	3,163,900	3,169,609	Receptor protein kinase PERK1	*ver/ph_11.1*	[48]
VIT_11s0016g03900	11	3,182,349	3,186,809	AAA-type ATPase	*ver/ph_11.1*	[48]
VIT_11s0016g03940	11	3,224,068	3,225,265	Heat shock transcription factor C1	*ver/ph_11.1*	[44, 46]
VIT_12s0035g02090	12	23,983,677	23,999,372	Leucine-rich repeat family protein	*ver/ph_12.1*	[48]
VIT_12s0035g02120	12	24,046,092	24,050,103	Unknown	*ver/ph_12.1*	[48]
VIT_14s0083g00620	14	22,672,469	22,675,655	NIK1 (NSP- interacting kinase 1)	*ver/ph_14.3*	[44, 46]
VIT_14s0083g00640	14	22,696,160	22,698,346	Constans 2 (COL2)	*ver/ph_14.3*	[44, 46]
VIT_14s0083g01030	14	23,320,331	23,341,036	putative MADS-box Fruitfull 2 (VviFUL2)	*ver/ph_14.3*	[44, 46, 48]
VIT_14s0083g01110	14	23,435,436	23,438,457	Brassinosteroid-6-oxidase	*ver/ph_14.3*	[44, 46]
VIT_14s0083g01160	14	23,527,926	23,532,692	COBRA protein	*ver/ph_14.3*	[48]
VIT_14s0083g01210	14	23,631,468	23,634,185	feronia receptor-like kinase	*ver/ph_14.3*	[44, 46]
VIT_14s0083g01220	14	23,647,671	23,648,618	feronia receptor-like kinase	*ver/ph_14.3*	[44, 46]
VIT_14s0068g00010	14	23,691,896	23,694,505	feronia receptor-like kinase	*ver/ph_14.3*	[44, 46]
VIT_14s0068g00030	14	23,710,282	23,713,253	feronia receptor-like kinase	*ver/ph_14.3*	[44, 46]
VIT_14s0068g00040	14	23,730,955	23,731,566	No hit	*ver/ph_14.3*	[44, 46]
VIT_14s0068g00050	14	23,741,203	23,741,804	No hit	*ver/ph_14.3*	[44, 46]
VIT_14s0068g00300	14	23,997,514	24,000,870	ABRC5	*ver/ph_14.3*	[48]
VIT_14s0068g00330	14	24,046,880	24,048,369	PTF1 (plastid transcription factor 1) TCP13	*ver/ph_14.3*	[44, 46]
VIT_14s0068g00640	14	24,438,706	24,450,994	Acetyl-CoA synthetase	*ver/ph_14.3*	[48]
VIT_16s0022g01650	16	15,243,820	15,246,842	Receptor serine/threonine kinase PR5K	*ver/ph_16.2*	[48]
VIT_16s0022g02230	16	16,240,572	16,248,680	Leucine-rich repeat receptor protein kinase EXS	*ver/ph_16.2*	[48]
VIT_16s0022g02340	16	16,470,141	16,475,595	fructokinase-2	*ver/ph_16.2*	[48]
VIT_16s0100g00350	16	17,248,816	17,261,155	ABC Transporter (VvTAP3 - VvABCB23)	*ver/ph_16.3*	[48]
VIT_17s0000g05020	17	5,637,669	5,644,801	Squamosa promoter-binding protein 6 (SPL6)	*ver/ph_17.1*	[44, 46]
VIT_17s0000g05050	17	5,659,282	5,660,704	COBRA-like protein 4	*ver/ph_17.1*	[48]
VIT_17s0000g05070	17	5,676,169	5,679,862	Phytochelatin synthetase	*ver/ph_17.1*	[44, 46, 48]
VIT_17s0000g05240	17	5,869,290	5,885,095	Nuclear transport factor 2 (NTF2)	*ver/ph_17.1*	[48]
VIT_17s0000g05580	17	6,213,229	6,221,132	Isopiperitenol dehydrogenase	*ver/ph_17.1*	[44, 46, 48]
VIT_18s0001g02000	18	2,438,485	2,442,668	Zinc finger (C2H2 type) family	*ver/ph_18.1*	[44, 46, 48]
VIT_18s0001g02540	18	2,802,829	2,805,078	ARR9 typeA	*ver/ph_18.1*	[44, 46]
VIT_18s0001g03580	18	3,389,546	3,393,993	Ubiquitin-fold modifier 1 precursor	*ver/ph_18.1*	[48]
VIT_18s0001g03670	18	3,422,279	3,424,214	Zinc finger (C2H2 type) family	*ver/ph_18.1*	[44, 46]
VIT_18s0001g04340	18	3,822,948	3,829,597	Glycine hydroxymethyltransferase	*ver/ph_18.1*	[48]
VIT_18s0001g04680	18	3,938,582	3,956,444	RPG related protein 1 RR1	*ver/ph_18.1*	[44, 46]
VIT_18s0001g06430	18	4,806,981	4,808,947	Homeobox-leucine zipper protein ATHB-6	*ver/ph_18.1*	[44, 46, 48]
VIT_18s0001g07090	18	5,290,562	5,293,561	Unknown protein	*ver/ph_18.1*	[48]
VIT_18s0001g12840	18	10,940,330	10,945,165	ADP-glucose pyrophosphorylase large subunit CagpL2	*ver/ph_18.2*	[48]
VIT_18s0001g13010	18	11,126,023	11,129,236	Mitogen-activated Protein Kinase (VvMPK11)	*ver/ph_18.2*	[48]
VIT_18s0001g13200	18	11,256,653	11,261,569	Cytokinin dehydrogenase 5 precursor	*ver/ph_18.2*	[44, 46]
VIT_18s0001g14130	18	12,179,540	12,181,647	Zinc finger (C2H2 type) family	*ver/ph_18.2*	[44, 46]
VIT_18s0001g14360	18	12,337,145	12,340,985	Tubulin beta-1 chain	*ver/ph_18.2*	[44, 46]
VIT_18s0001g14440	18	12,432,955	12,439,459	Molecular chaperone DnaJ	*ver/ph_18.2*	[44, 46]
VIT_18s0001g14450	18	12,453,766	12,456,583	Ferredoxin:nadp+ Oxidoreductase PETH	*ver/ph_18.2*	[44, 46]
VIT_18s0001g15720	18	13,856,940	13,861,020	Leucine Rich Repeat receptor-like kinase	*ver/ph_18.2*	[44, 46, 48]
VIT_18s0001g15730	18	13,865,318	13,866,466	Dof zinc finger protein DOF3.5	*ver/ph_18.2*	[48]
VIT_18s0076g00330	18	14,494,814	14,503,181	Basic Leucine Zipper Transcription Factor (VvbZIP50)	*ver/ph_18.2*	[48]
VIT_18s0076g00310	18	14,550,818	14,563,944	Translation initiation factor eIF-5B	*ver/ph_18.2*	[48]

^a^For each candidate physical position on the grape genome assembly as well as annotation is provided. Genes shown in the tables are selected by following slimGOs: GO:0000166, GO:0003676, GO:0003677, GO:0003682, GO:0003700, GO:0005102, GO:0005634, GO:0007154, GO:0007165, GO:0007275, GO:0009653, GO:0009719, GO:0009791, GO:0009908, GO:0016301, GO:0030154, GO:0038023, GO:0040007

^b^Meta-QTL under which the candidate was found

^c^Reference of the transcriptomic study in which the candidate was found to be differentially expressed during veraison

As expected, some of the proposed candidates were previously pointed out by QTL studies or by analysis of transcriptomic profiles. However, integration of available QTLs genetic data, by meta-QTL analysis, with transcriptomic data has allowed prioritization of the huge number of candidates, reducing by about 20 and 10 times the genes proposed so far by either only genetic or transcriptomic approaches (Fig. 2). Among these genes we expect to be included, according to all available molecular information, those controlling the grapevine veraison transition.

## Discussion

A classical way to dissect the genetic determinism of grape phenology has been QTL studies [22–26, 53, 58]. However, QTLs mapping often provides inconsistent results among studies, and huge genomic locations. A big advantage can derive from meta-analysis, which offers stronger evidence than individual studies, by revealing regions robustly associated with traits in multiple environments and genetic backgrounds [29, 95]. This approach has been already successfully exploited to improve and validate QTLs in several species, allowing insights into the genetic architecture of complex traits and paving the way for fine mapping and gene cloning [32, 34–37]. With this aim a genetic consensus map was built from 39 available simple sequence repeats (SSR)-based maps, including 3130 markers. By looking at marker distribution we observed they were not regularly spread along the chromosomes, but tended to concentrate in the middle regions, even though a good correlation was found with original maps. This is not surprising, reflecting a similar trend to original maps, due to suppression of recombination in centromeric regions. Other consensus maps already reported this drawback [35, 96]. Moreover, genetic positions of markers on the consensus arose from positions of shared markers according to the BioMercator software procedure [97], and were not based on recombination, since original genetic data are unfortunately not available from original maps. We fully agree that QTL meta-analysis would gain power and precision if raw genotypic and phenotypic data were made available. Recent advances in markers technology, with development of the next generation sequencing-based genotyping by sequencing (GBS) technology in particular, have given a strong impulse to plant genotyping, and QTL studies now rely more on dense single nucleotide polymorphism (SNP) maps. However, unshared markers do not allow for a direct genetic comparison of mapped QTLs, but require an indirect comparison through anchoring to the genome assembly. The distribution pattern of QTLs on chromosomes differs strongly between genetic and physical maps [96]. Therefore, integration directly at genetic level could aid the improving of QTL location through co-location and meta-analysis, when feasible. Further comparisons can be then undertaken to newly generated QTLs relying on high throughput SNP maps, following anchoring to the genome assembly. Taking all this into account, we concluded that the consensus map we built constitutes a valuable reference, especially to the aim of integrating available genetic information, from related QTL studies. Moreover, it will also provide a valuable instrument to enquire co-location with newly generated QTLs relying on dense SNP maps.

Taking advantage of this tool we have provided a compendium of all available QTL information that can be integrated at genetic level. Interestingly QTLs plotting revealed extensive co-locations across studies for each of the phenology related traits, besides downy mildew resistance, powdery mildew resistance, anthocyanin, drought stress, fertility, water use efficiency and growth, as well as for some berry and seeds related traits. However, studies addressing phenology are still few, negatively affecting the number of studies supporting each of the co-located QTLs. *R*^*2*^ values of plotted QTLs, beside their distribution, suggest a highly polygenic nature for phenology related traits, with several QTLs involved, each of small effect, differently from other traits like pathogen resistance, seeds related traits and colour, all showing a more oligogenic architecture. More in detail, concerning veraison time four main regions located on LG 1 and 2 have so far emerged consistently. Interestingly, plotting on a unique consensus map of QTLs also allows inspection of co-location across traits and categories, which is especially relevant for complex traits. In this way QTL meta-analysis also allows genetic correlation among traits to be investigated [35, 92, 98, 99]. In a previous work a second round meta-QTL analysis was proposed for seed yield QTLs and co-located yield associated QTLs in rapeseed, which allowed “indicator” meta-QTLs contributing to the complex trait crop yield to be defined [100]. Indeed, QTL co-localization can be due to tight-linkage of QTL/genes playing different functions, but could also arise from pleiotropism. When pleiotropy is likely, it would also justify meta-analysis across traits, to further reduce the number of candidates [100]. Veraison time is expected to be strictly related to other phenological stages [9]. Accordingly, tests on the previously mentioned regions on LG 1 and LG 2 confirmed that, at least in some cases, comparable results are achieved when only veraison or all co-located phenology related traits are considered for the meta-analysis (see *ver_2.1 and ver/ph_2.1* as an example). We therefore also attempted a similar approach for veraison QTLs co-located with other phenology QTLs, finally identifying a number of regions, of which the most relevant were those located on LGs 14, 16 and 18. However, we are aware that these rely on the pleiotropic assumption, which could be not always satisfied. A recent QTL study based on a GBS SNP map also addressed the mapping of veraison time [101]. That study mainly aimed to discover and map stable QTLs across environments. A veraison QTL mapping on LG 16 between 5 and 24 cM, which corresponded to the region between 2 and 16 Mbp, was found, but was not consistent across environments. Interestingly, it partially overlapped the *ver/ph_16.2* meta-QTL we derived here starting from a veraison QTL and its co-location to a flowering-veraison interval QTL. Beside the detailed analysis of phenology traits we have undertaken, our compendium now provides a useful tool for the inspection of co-location and meta-analysis for further traits in a similar way.

Transcriptomic studies have been also widely applied to characterize molecular changes associated to the onset of ripening, revealing, first of all, a massive transcriptomic rearrangement at veraison time [44, 49]. Among others, genes triggering such transition are expected to modulate their expression at this stage, although alternative regulative mechanisms cannot be excluded. We thus mined available transcriptomic profiles to i) identify the timing of such massive change, ii) select genes differentially expressed during this time in more varieties. Then, beside inspection of positional candidates underlying meta-QTLs, we propose to also integrate information about differential expression at veraison time, in order to prioritize candidates.

On LG 1 a veraison time QTL was previously mapped [25]. A more recent study [26] also mapped a QTL for veraison at this location, which allowed us to define the *ver_1_1* meta-QTL. Flowering QTLs consistently overlapped at same location [22, 58] suggesting a possible control of veraison time through regulation of flowering time. Accordingly, candidates for the flowering time control mapped under this meta-QTL, like the *PFT1 (phytochrome and flowering time 1)* gene or a *CONSTANS-like* gene both controlling the photoperiodic flowering pathway in *A. thaliana* [102, 103]. Even though a possible impact of the genetic control of flowering on veraison time would reduce the relevance of candidates found by our transcriptomic approach, integration of transcriptomic data allowed to pinpoint 14 candidates, among which the VvRAV1 transcription factor, belonging to the plant-specific *RAV (RELATED TO ABI3 AND VP1)* family. In Arabidopsis, RAV1 was shown to act as negative regulator of both development and flowering, probably in complexes with other co-repressors [104–106]. Interestingly, some members of this gene family were shown to modulate developmental transitions in response to temperature [107]. Moreover RAV1 was also shown to be negatively regulated by brassinosteroid and abscisic acid [104, 108], both hormones modulated at the onset of veraison time [1].

On LG 2 meta-QTL analysis of overlapping veraison QTLs allowed 3 main regions to be spotted. In the first of these regions flowering QTLs were also plotted [22], again supporting a possible regulation of veraison time through flowering, even though no genes controlling flowering time where found under this locus. Interestingly, the orthologous of the Arabidopsis YABBY1/FIL transcription factor, which directly activate the AtMYB75, a key regulator of anthocyanin biosynthesis [109], was found among candidates selected by the integration of expression data. Moreover, by looking at other functional categories possibly related to veraison time, a gene encoding for a vacuolar invertase 2, key enzyme of sugar metabolism in fruits during ripening [110], a stay-green protein 1 gene related to a gene shown to be involved in ripening in tomato [111], beside two pectin methylesterase inhibitor (PMEI) genes, were found as differentially expressed. These last belong to a gene family previously characterized in grape [112]. Their function is supposed to inhibit pectin methylesterase activity in pectin degradation, and may play a role in the beginning of ripening by regulating initial events such as softening and loss of turgor [113]. Interestingly, network analysis of gene expression profiles during berry ripening revealed PMEI among genes likely involved in triggering the major transcriptome reprogramming that occurs at veraison [44]. Within *ver_2.2* meta-QTL, the most notable candidate considering both positional and expression data was the VvNAC13 transcription factor. This gene belongs to a wide family of transcription factors in grapevine [114]. Interestingly members of this family in tomato are involved in ethylene biosynthesis, reception and signalling during ripening [115]. Moreover, they were also already suggested as playing a crucial role in berry transcriptome modulation associated to veraison, according to network analysis of berry expression profiles [44]. However, in the same region, a gene encoding an atypical pseudo-response regulator (APRR2), involved in the circadian clock mechanism and contributing to fruit pigmentation and ripening in tomato [116], as well as two 1-aminocyclopropane-1-carboxylate oxidases, taking part in ethylene biosynthesis and ripening were also selected by our approach and represent promising candidates. Lastly, a cluster of Myb genes locates within *ver_2.3* meta-QTL interval. These genes have previously been extensively characterized for their involvement in the transition to berry ripening, by regulating the accumulation of anthocyanins in the berry skin [93, 94]. This finding, thus, supports our approach, even though these genes are unlikely to be themselves the early triggers of ripening onset.

Other genomic regions were also proposed by previous studies for the genetic control of veraison time [22–26], among which the most relevant were mapping on LG 14, 16 and 18. By considering overlapping with other phenology related QTLs, followed by integration of transcriptomic data, we also selected candidates for these regions. The *ver/ph_14.3* meta-QTL was computed from overlapping veraison QTL and flowering QTLs [23, 25, 58], and was accordingly highly enriched in candidates playing a role in the flowering transition control or fruit ripening, among which the most notable are Constans 2 (COL2), the feronia receptor-like kinase, a gene encoding a Brassinosteroid-6 oxidase, a gene encoding a COBRA protein and the putative MADS-box FRUITFULL 2. Interestingly this last gene was recently shown to also contribute to modulate the onset of ripening in tomato at early fruit development, beside its involvement at later ripening stages [117]. A QTL previously mapped on LG 16 and explaining a large part of the genetic variance in the corresponding mapping population [26] partially co-localized to QTLs for the derived trait flowering-veraison interval [22, 23] and to the genomic region involved in veraison recently identified by the GBS-SNP map previously discussed [101]. According to our strategy, the original interval was reduced to two regions of about 3.3 Mbp overall, including 15 transcriptomic candidates. Interestingly, more recently, the SSR marker UDV052, mapping under the *ver/ph_16.3* meta-QTL close to the two candidates ABC transporter and an ERF transcription factor (19.1 Kbp and 56.9 Kbp respectively), was shown to be significantly associated to the early phenotype in a collection of different varieties, thus supporting our approach [118]. Lastly, three different veraison QTLs were mapped on LG18 [26]. Two of them partially co-located with flowering QTLs from an independent study, and one of them was overlapping also with a QTL for the flowering-veraison interval [23, 58]. Under the derived meta-QTLs, *ver/ph_18.1* and *ver/ph_18.2,* spanning a still large region, we selected 74 transcriptomic candidates among which 19 were encoding proteins involved in regulation of gene expression, signalling or development. Candidates involved in carbohydrate metabolism, including especially a hexose (HT2) and a sucrose transporter (SUT2-2), putatively modulating sucrose signalling, or candidates encoding for genes for cell wall degradation (like a glucanase and a galactosidase, as examples), were also among those selected.

## Conclusions

By building a grape consensus genetic map anchored to the genome assembly a comprehensive overview about genomic distribution of several QTLs from published studies and their co-location both inside traits as well as across related traits was provided. Extensive co-localization was evident especially for phenology related traits. Four veraison meta-QTLs located on LG 1 and 2 were found. Moreover several additional meta-QTLs, computed from co-localization of veraison QTLs with alternative phenology related QTLs, were derived, among which most relevant on LG 14, 16 and 18. Integration of meta-QTLs with expression data from prior transcriptomic studies allowed to select a set of 272 candidate genes for the genetic control of the veraison transition, reducing by about 20 and 10 times the genes proposed so far by either only genetic or transcriptomic approaches. Among these candidates 78 genes were involved in regulation of gene expression, signal transduction or development. Specific relevant candidates according to their annotation have been discussed. Further studies can now test and eventually validate the putative involvement of these candidates in the genetic control of the veraison transition during berry development.

## Methods

### Collection of QTL studies and QTLs data

All published QTL experiments on grapevine were collected, mainly by using the public database PubMed (https://www.ncbi.nlm.nih.gov/pubmed/) and searching for “grape” and “QTL”. QTL experiments were selected if relying on genetic maps including shared SSR markers and if all required information for further analysis were available (Additional file 1). Individual genetic linkage map including marker names and position in cM were transcribed from all experiments. Consensus map was selected, when this was provided. Parental maps were included only if consensus maps were not available (see Additional file 1 for more details). Data about mapped QTLs were also transcribed, in particular start and end position of the QTL, confidence interval in cM, peak of the QTL in cM, QTL associated variance explained value (*R*^*2*^) and the size/type of the population that was used for mapping the QTL. All QTLs were included, independently of their phenotypic scoring system, score thresholds, LOD/variance values or years of observation. Details on original QTL short names, as well as a short description, are given in Additional file 2. Only for the veraison trait, on which this work is mainly focused, further details about the different phenotypic scoring systems in the different original publication have been collected (Additional file 19). For all other QTLs we refer to the original publication for more details about the phenotypic scoring. QTLs were attributed to eight main categories including related traits, to aid storage and further studies. All markers and QTLs information were properly formatted to be imported into BioMercator v4.2 software [87].

### Building of a grapevine consensus map

Name of the markers in each map were manually curated in order to correct misspellings and find synonyms. Indeed, to combine the individual maps into a consensus map, markers' name requires to be consistent. Each map file was imported in BioMercator v4.2 [87] and each linkage group was oriented according to the reference map published in Doligez et al., 2006 [88]. Linkage groups that did not share at least two markers with others were removed from the analysis, since they could not be properly oriented. This led to a different number of input maps for each linkage group depending on the chromosome. InfoMap command in the software was used to evaluate markers order and consistency between each pair. In case of inversions, occurrence of inverted markers in all the maps was evaluated and the less represented marker across all maps was removed to retain most frequent common marker. When no more inversions were left, the command ConsMap was used to build the consensus map in a single step chromosome by chromosome, without providing any reference.

### Anchoring to the grapevine genome by in silico mapping of GCM markers

Grape Consensus Map (GCM) markers’ sequences were downloaded from original publications and blasted against the 12X.v2 assembly of the grapevine genome using the website https://urgi.versailles.inra.fr/blast/. An anchor map was created including all univocally mapping GCM markers with corresponding base pairs positions. The anchor map was uploaded to BioMercator v4.2 and the option “New genome version” was used to anchor the GCM to the grapevine genome from the .gff3 file (https://urgi.versailles.inra.fr/Species/Vitis/Annotations). This allows recovering of physical intervals for any feature (like QTLs or meta-QTLs), through BioMercator using a software internal formula (Yannick De Oliveira, personal communication).

### QTL projection

Each QTL was associated to the genetic map were it was originally mapped. The command QTLProj in the BioMercator v4.2 software was applied to project the QTLs of the component maps to the consensus map. The command performs a homothetic projection of the original QTL to the consensus map based on flanking markers and using a scaling rule. This is applied only when flanking markers are found where the ratio of the distance of these markers to the confidence interval of the QTL that is being projected is not reduced by a factor greater than 0.25. Default options were kept for the analysis. Consensus QTL maps were extracted for each trait and manually inspected for QTLs co-location across populations. All regions spanned by QTLs for a same trait mapped in different mapping populations were recorded. Significance of QTLs co-localization was calculated as described in [92].

### QTL meta-analysis

The meta-QTL analysis was performed by using the QTLClust command in BioMercator v4.2 software when at least two overlapping QTLs belonging to the same trait were found. Redundant QTLs, that is, QTLs on same position from same study, which could overestimate the effect of that QTL in the analysis were pruned retaining that with highest *R*^*2*^ prior the analysis [35]. The meta-analysis was executed selecting the Veyrieras algorithm [28]. Optimal number of meta-QTLs explaining overlapping QTLs was statistically determined by choosing the most likely model, as computed by the software according to five different tests. Indeed the software performs the clustering of the input overlapping QTLs for a trait and allows determining the most likely number of meta-QTLs, calculating models for as many QTLs up to the number of the input QTLs and providing values for each model for five different criterion: the AIC (Akaike information criterion), the AICc, the AIC3, the BIC (Bayesian information criterion) and the AWE (average weight of evidence). Best model was selected as the one minimizing values for the highest number of criterion which represents the optimal number of clusters that best explain the observed QTL distribution. MQTLView command allows to graphically represent the meta-QTLs according to the selected model. A second round of meta-QTL analysis was performed as described in [35] by merging veraison QTLs and other overlapping phenology related traits for meta-QTL analysis.

Physical intervals for each meta-QTL were computed as previously explained through anchoring to grape genome assembly 12X.v2 in the BioMercator v4.2 software and underlying candidate genes retrieved including their functional annotation according to CRIBIv1 annotation (http://genomes.cribi.unipd.it/gb2/gbrowse/public/vitis_vinifera_v2/). Gene ontology annotations were retrieved by using the *getBM* function of the *Bioconductor biomaRt* (2.38.0) package. *Vitis vinifera* Ensembl database was used and candidate genes were annotated with GO slim accessions.

### Transcriptomic data integration

The grapevine expression atlas [49] was used to retrieve expression in different grape organs and exclude candidate genes never expressed in berry, rachis or seeds. RNA-Seq gene expression data along berry development were retrieved from three studies [44, 46, 48]. Ninety-nine berry RNA-Seq profiles for the cultivars Pinot Noir collected in triplicates in the years 2012, 2013 and 2014 around the time of veraison [48] were retrieved. Genes with FPKM (fragments per kilobase of exon model per million reads mapped) values lower than 1 in at least 2 replicates at all time-points were considered as never expressed and removed from the dataset. Expression of early “biomarkers” of veraison transition [48] was inspected at early time points during berry development before veraison to identify the interval in each year when the transcriptomic rearrangement associated to veraison first occurs. Genes showing highest modulation in their expression across this interval in at least 2 years were selected by inspecting FPKM values and considered as transcriptomic candidates to be joined to genes differentially expressed across veraison in all varieties as defined in [44, 46]. Finally transcriptomic candidates positioned under meta-QTLs were selected.

## Supplementary information

**Additional file 1.** List of publications including grapevine QTL studies selected as suitable for integration of QTLs data. For each publication are reported reference of the genetic map used in the original publication, details about the cross population used for QTL mapping and the total number and categories of QTLs included in the analysis.

**Additional file 2.** List of phenotypes scored in the selected QTL studies, grouped according to the study. The list includes the original QTL short name attributed in the reference to each phenotype, as well as a short description. The main trait for which the phenotype was considered to be a descriptor and the trait category are also indicated for each phenotype.

**Additional file 3.** Overview of selected grapevine QTLs included in the analysis. (a) Number of QTLs for each trait, shown separately for the 8 different trait categories. (b) Number of QTL studies addressing each of the traits, shown separately for each category. Studies addressing more traits are repeatedly count in each category, so plotted numbers of QTLs studies for each category is redundant. The number of unique studies for each category is shown in brackets. Colour code for each trait is given in the legend table.

**Additional file 4.** The grape consensus genetic map built from 39 grape linkage maps. The table includes physical marker position if available, for anchoring to the grapevine genome assembly.

**Additional file 5.** Graphical overview of the consensus genetic map.

**Additional file 6.** Number of maps used for the construction of each consensus linkage group.

**Additional file 7.** Spearman’s rank correlation values of each pairwise comparison between markers' order of each single component map and with the consensus map. References for each single component map are reported in Additional file 6.

**Additional file 8.** Consensus QTL maps for each of the 34 traits built by projection onto the consensus map of QTLs from the selected 47 QTL studies. Each map is provided in a separate sheet and for each the whole and short QTL name, as well the genetic position information and physical position information obtained through marker anchoring of the map to the genome are given. Details about QTL short names abbreviations are in Additional file 2.

**Additional file 9.** Circular plots of consensus QTL maps built by plotting of QTLs on the consensus map. Circular plots are grouped according to categories: (a) abiotic stress response, (b) cluster related traits, (c) berry morphology, (d) berry metabolites, (e) pathogen resistance, (f) seed related traits, (g) vegetative traits. QTLs are shown in the internal side of each chromosome. Genetic regions spanned by QTLs confirmed in independent population are highlighted by a bar on the outer side of the chromosomes. Colour code for each trait is given in the legend table.

**Additional file 10.** List of QTLs showing at least partial overlapping with similar QTLs from independent studies. Whole and short QTL names, as well as the genetic position information and physical position information obtained through marker anchoring of the map to the genome are given. Details about QTL short names abbreviations are in Additional file 2.

**Additional file 11.** Meta-QTLs calculated from veraison QTLs overlapping with other phenology QTLs on LG1 and LG2 providing meta-QTLs in high agreement to meta-QTLs calculated only from veraison QTLs.

**Additional file 12. **Lists of candidate genes in *ver* meta-QTLs intervals with the corresponding genomic positions derived by anchoring to the genome and CRIBIv1 annotation. GO annotation was included only for genes with GOs related to expression regulation, signalling or development (GO:0000166, GO:0003676, GO:0003677, GO:0003682, GO:0003700, GO:0005102, GO:0005634, GO:0007154, GO:0007165, GO:0007275, GO:0009653, GO:0009719, GO:0009791, GO:0009908, GO:0016301, GO:0030154, GO:0038023, GO:0040007) representing most relevant positional candidates for veraison regulation according to functional annotation.

**Additional file 13. **Lists of candidate genes in *ver/ph* meta-QTLs intervals with the corresponding genomic positions derived by anchoring to the genome and CRIBIv1 annotation. GO annotation was included only for genes with GOs related to expression regulation, signalling or development (GO:0000166, GO:0003676, GO:0003677, GO:0003682, GO:0003700, GO:0005102, GO:0005634, GO:0007154, GO:0007165, GO:0007275, GO:0009653, GO:0009719, GO:0009791, GO:0009908, GO:0016301, GO:0030154, GO:0038023, GO:0040007) representing most relevant positional candidates for veraison regulation according to functional annotation.

**Additional file 14.** Meta-QTLs calculated from 28 overlapping QTLs from 5 independent studies for the well characterized anthocyanin colour trait mapping at the colour locus on LG2.

**Additional file 15 **Lists of candidate genes in the 7 *anthocyanin* meta-QTLs intervals identified by meta-QTLs analysis as reported in Additional file 14. Positional information were derived by map anchoring to the genome and CRIBIv1 annotation. This list includes the *VvMYBA1* and *VvMYBA2* genes well known for involvement in anthocyanin biosynthesis and colour determination in grape, thus supporting the meta-QTLs approach applied in this study.

**Additional file 16.** Number of differentially expressed biomarker genes obtained by comparison of expression levels at each time point before visual veraison and next one, until visual veraison. Each time point is expressed as days before visual veraison. Number of early “biomarkers” of veraison transition as defined in [48] found as differentially expressed across intervals are given. The interval across which the highest number of biomarkers was differentially expressed represents the timing when early transcriptomic rearrangement associated to the veraison starts to occur in each year.

**Additional file 17.** Whole list of transcriptomic candidates derived by studies of expression profiles along berry development [44], [46], [48] including positional information and functional annotation. Information about the original study in which they were selected and about their eventual prioritization through network analysis of expression profiles (“switch genes”) or selection as “biomarkers” according to profile analysis in original studies is included.

**Additional file 18.** List of 272 genes selected by integrating the list of transcriptomic candidates and positional information from the meta-QTL analysis. Positional information (Chr and position) derived by map anchoring to the genome, functional annotation and information about the transcriptomic study in which each candidate was first reported are included for each selected candidate in the table. Table 5 represents a subset of this table, including, among these candidates, those involved in signalling, development or regulation of gene expression.

**Additional file 19.** Phenotypic scoring details for selected veraison QTLs used in the work. Details about the phenotypic scoring have been collected from original publications and reported here alongside with QTL short name, QTL description and the reference publication.

## Data Availability

All genetic and QTL data used in this article are referenced in Table 1 and Additional file 1. The datasets including the consensus genetic map and all consensus QTL maps compiled and supporting the conclusions of this work are included within the article and its additional files. Transcriptomic datasets used referred to the atlas grapevine transcriptomic dataset performed by microarray [49], the grapevine berry RNA-Seq data for red and white skinned varieties [46], and the grapevine berry RNA-Seq data on Pinot noir [48], and are available in a Minimum Information About a Microarray Experiment (MIAME)-compliant database (Gene Expression Omnibus) at the National Center for Biotechnology Information, with the GSE36128, GSE62744 and GSE62745 and GSE98923, respectively.

## Abbreviations

Ac: Total acidity
AIC: Akaike information criterion
At: Tartaric acid
AWE: Average weight of evidence
BB: Bud break
B-B: February–budbreak/Bud
B-F: Budbreak–flowering/Flo
BIC: Bayesian information criterion
Bp: Basepair
Bpc: Brixper cluster
Chr: Chromosome
CI: Confidence interval
cM: centiMorgan
Cma: Fruit maturation period
FB: Flowering beginning
FBL: Time of full bloom
FPKM: Fragments per kilobase of exon model per million reads mapped
F-R: Flowering ripening interval
FS: Start of flowering
FT: Flowering time
F-V: Flowering-veraison interval
Fw: Flowering
GBS: Genotyping by sequencing
GCM: Grape Consensus Map
GO: Gene ontologies
LG: Linkage group
Ma: Malic acid
QTL: Quantitative trait loci
R: Ripening
RDA: Ripening date
Rp: Ripening
RT: Ripening time
SNP: Single nucleotide polymorphism
Sp: Sprouting
ssc: solubile solids concentration
SSR: Simple sequence repeats
ta: total acid
Tar/Ma: Ratio of tartaric acid to malic acid
Tss/Ac: Ratio of total soluble solids to total acidity
VB: Veraison beginning
VE: Veraison end
*ver*: veraison meta-QTLs
*ver/ph*: meta-QTL from veraison QTLs and other phenology related overlapping QTLs
VP: Veraison period
Vr: Veraison
V-R: Veraison ripening interval
Vr-Rp: Veraison-ripening period
VT: Veraison time
