# Supplementary material for: Selection of candidate genes controlling veraison time in grapevine through integration of meta-QTL and transcriptomic data
Source: BMC Genomics. 2019 Oct 15;20:739. doi: 10.1186/s12864-019-6124-0 (PMC6794750; doi:10.1186/s12864-019-6124-0)
Supplement: Supplementary file 3 — Additional file 3. Overview of selected grapevine QTLs included in the analysis. (a) Number of QTLs for each trait, shown separately for the 8 different trait categories. (b) Number of QTL studies addressing each of the traits, shown separately for each category. Studies addressing more traits are repeatedly count in each category, so plotted numbers of QTLs studies for each category is redundant. The number of unique studies for each category is shown in brackets. Colour code for each trait is given in the legend table. [file 12864_2019_6124_MOESM3_ESM.pdf]

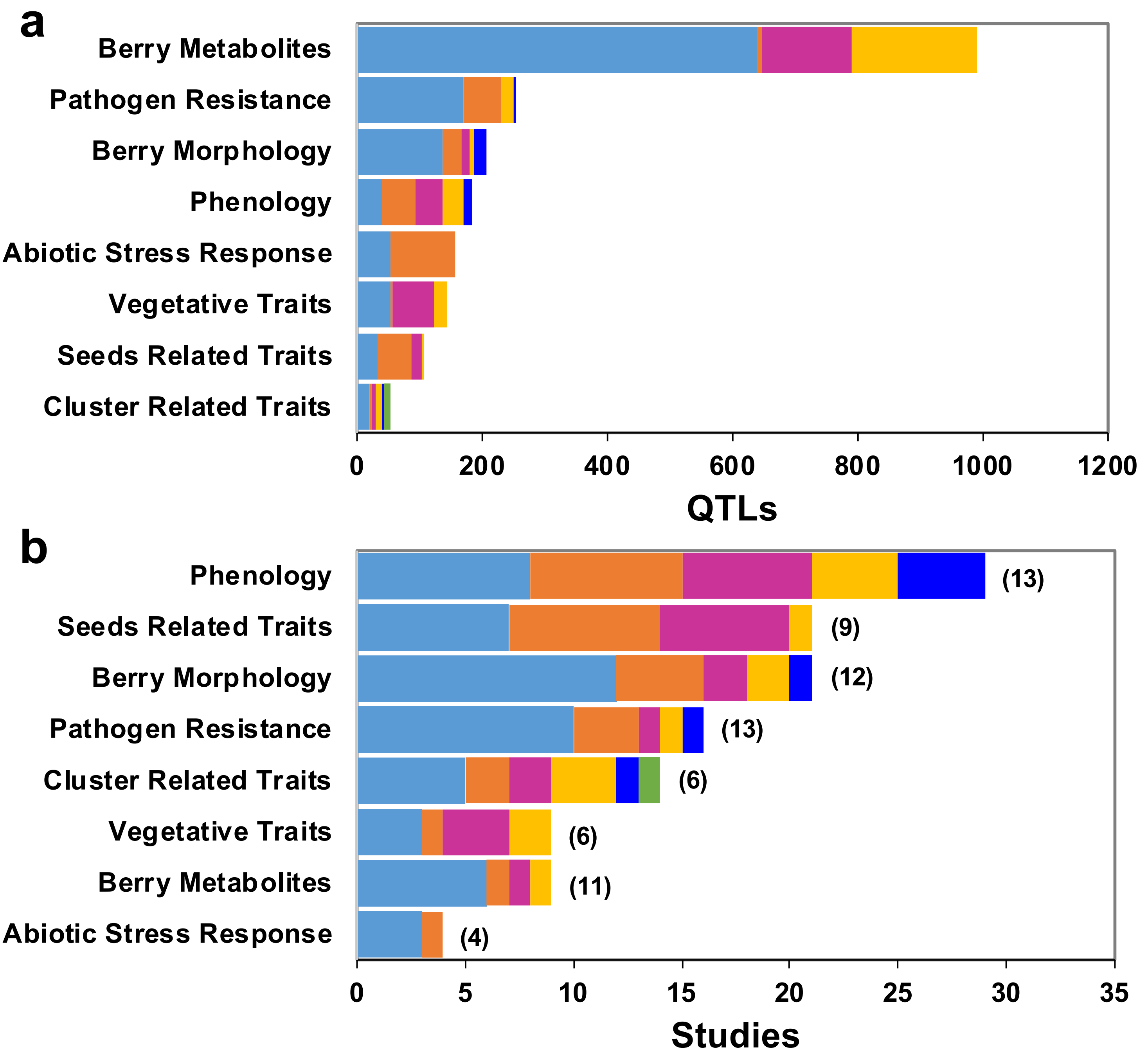


| Abiotic Stress Response | Berry Metabolites | Berry Morphology | Cluster Related Traits | Pathogen Resistance | Phenology | Seeds Related Traits | Vegetative Traits |
|---|---|---|---|---|---|---|---|
| Drought Stress | Anthocyanins | Berry Weight | Fertility | Downy Mildew Resistance | Ripening Time | Seed Number | Growth |
| Chlorosis | Terpenols | Berry Firmness | Berry Number | Powdery Mildew Resistance | Veraison Time | Seed Weight | Leaf Morphology |
| | Seed And Skin Tannins | Berry Diameter | Peduncle Length | Phylloxera Resistance | Flowering Time | Seed Percent Dry Matter | Water Use Efficiency |
| | Flavonol | Berry Volume | Cluster Architecture | Black Rot Resistance | Interval | Seed Response To Ga | Leaf Area |
| | | Berry Response To Ga | Cluster Weight | Botrytis Resistance | Budburst | | |
| | | | Cluster Response To Ga | | | | |
