# Supplementary figures and images for: Selection of candidate genes controlling veraison time in grapevine through integration of meta-QTL and transcriptomic data
Source: BMC Genomics. 2019 Oct 15;20:739. doi: 10.1186/s12864-019-6124-0 (PMC6794750; doi:10.1186/s12864-019-6124-0)

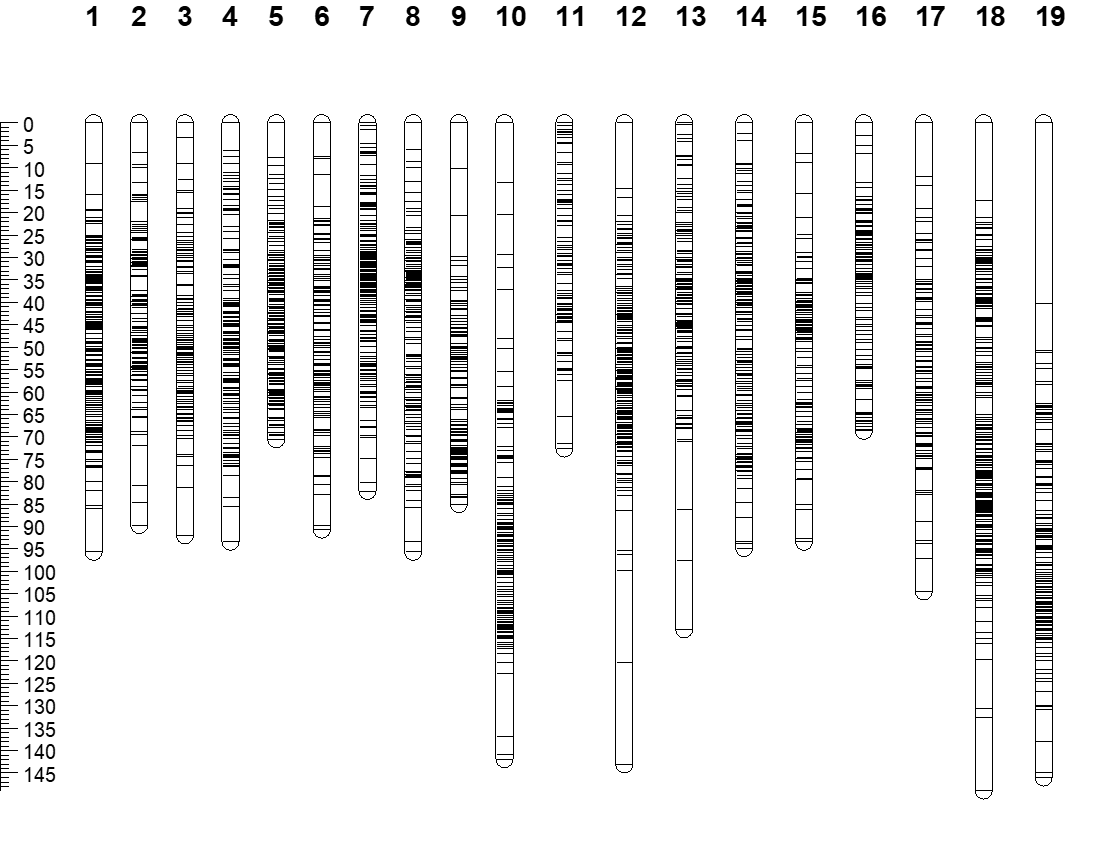

Supplement: Supplementary file 5 — Additional file 5. Graphical overview of the consensus genetic map. [file 12864_2019_6124_MOESM5_ESM.tif]

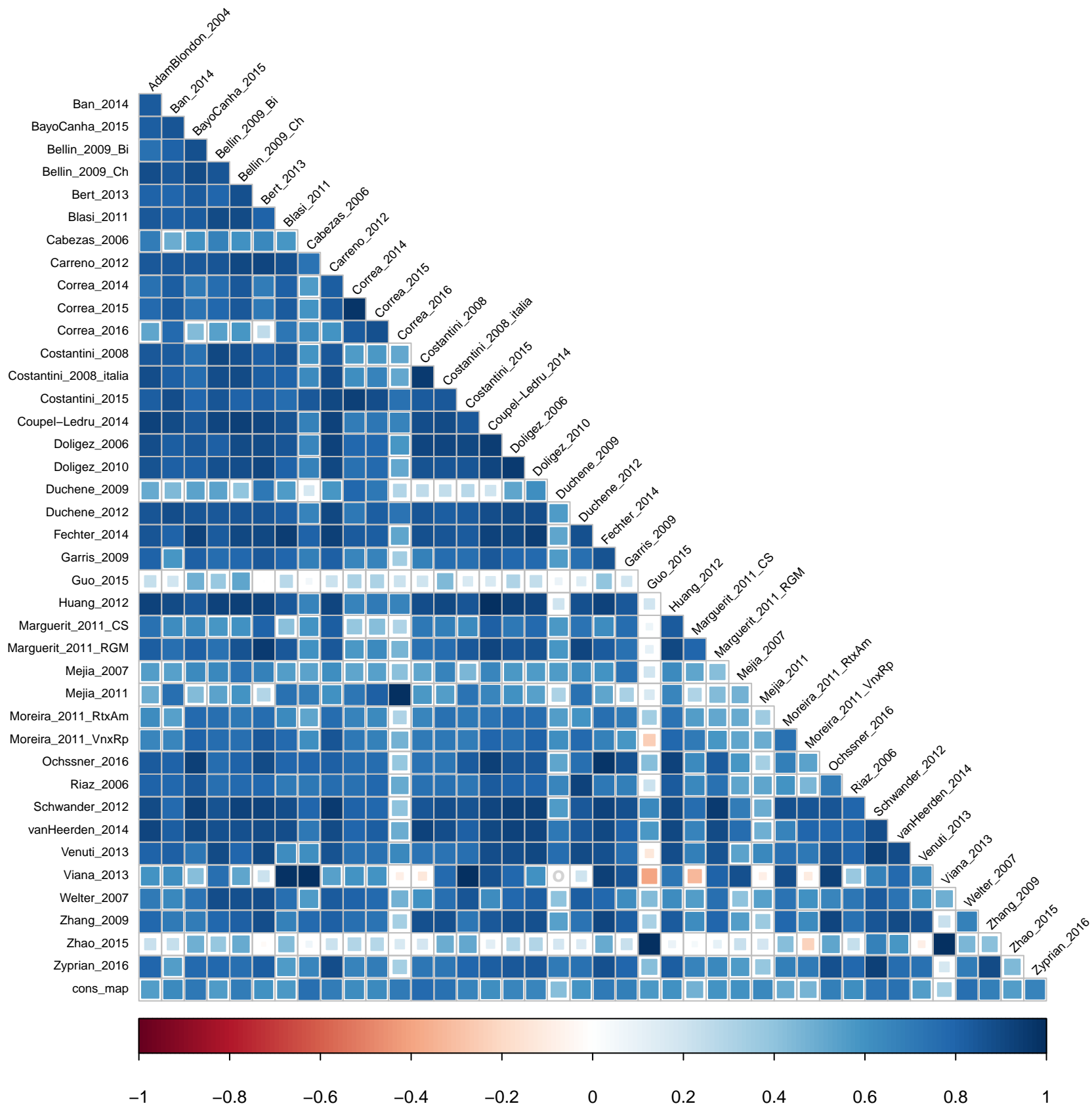

Supplement: Supplementary file 7 — Additional file 7. Spearman’s rank correlation values of each pairwise comparison between markers' order of each single component map and with the consensus map. References for each single component map are reported in Additional file 6. [file 12864_2019_6124_MOESM7_ESM.pdf]
