# Supplementary material for: Selection of candidate genes controlling veraison time in grapevine through integration of meta-QTL and transcriptomic data
Source: BMC Genomics. 2019 Oct 15;20:739. doi: 10.1186/s12864-019-6124-0 (PMC6794750; doi:10.1186/s12864-019-6124-0)
Supplement: Supplementary file 9 — Additional file 9. Circular plots of consensus QTL maps built by plotting of QTLs on the consensus map. Circular plots are grouped according to categories: (a) abiotic stress response, (b) cluster related traits, (c) berry morphology, (d) berry metabolites, (e) pathogen resistance, (f) seed related traits, (g) vegetative traits. QTLs are shown in the internal side of each chromosome. Genetic regions spanned by QTLs confirmed in independent population are highlighted by a bar on the outer side of the chromosomes. Colour code for each trait is given in the legend table. [file 12864_2019_6124_MOESM9_ESM.pptx]

## Slide 1
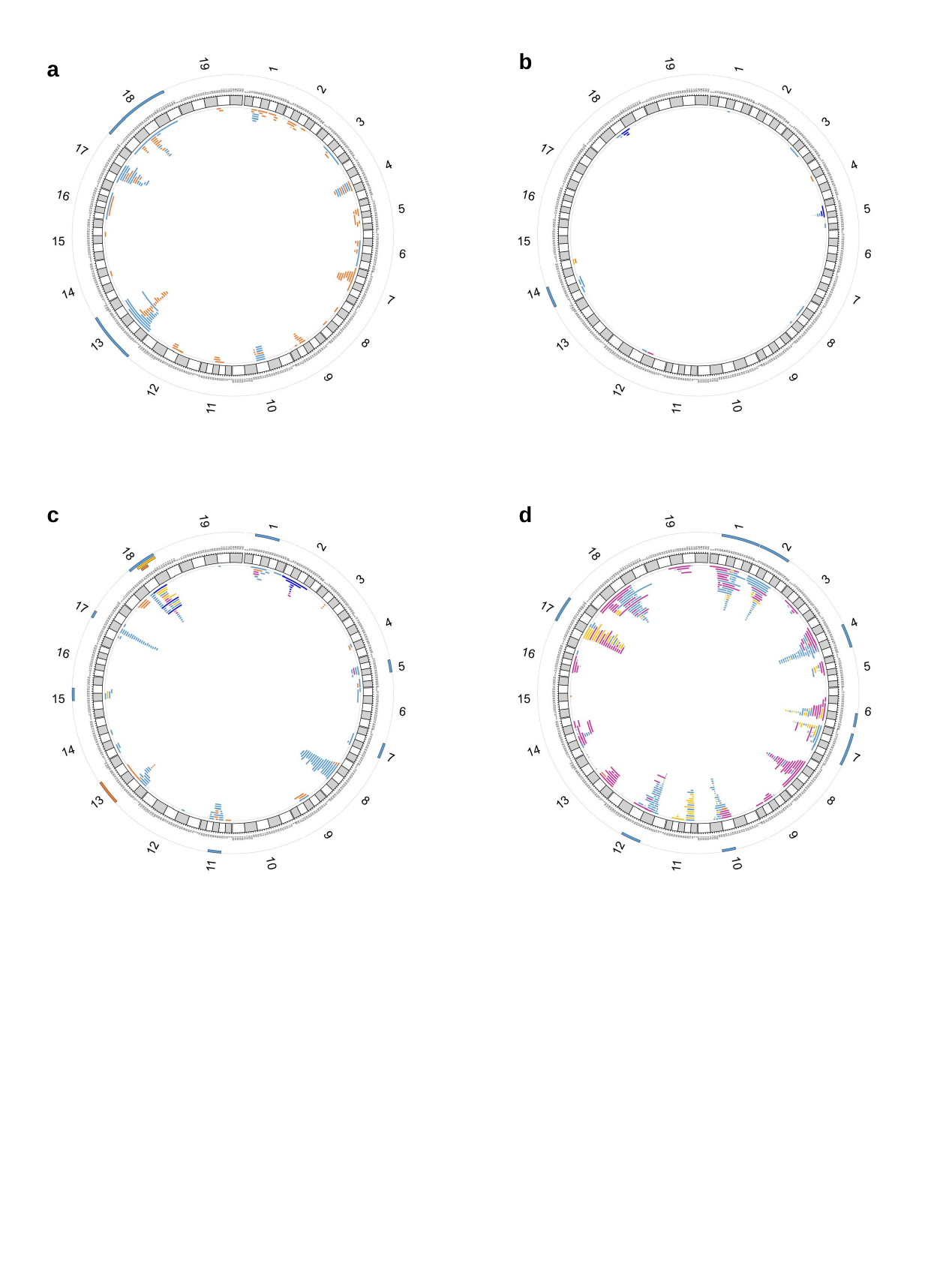

b
a
c
d

## Slide 2
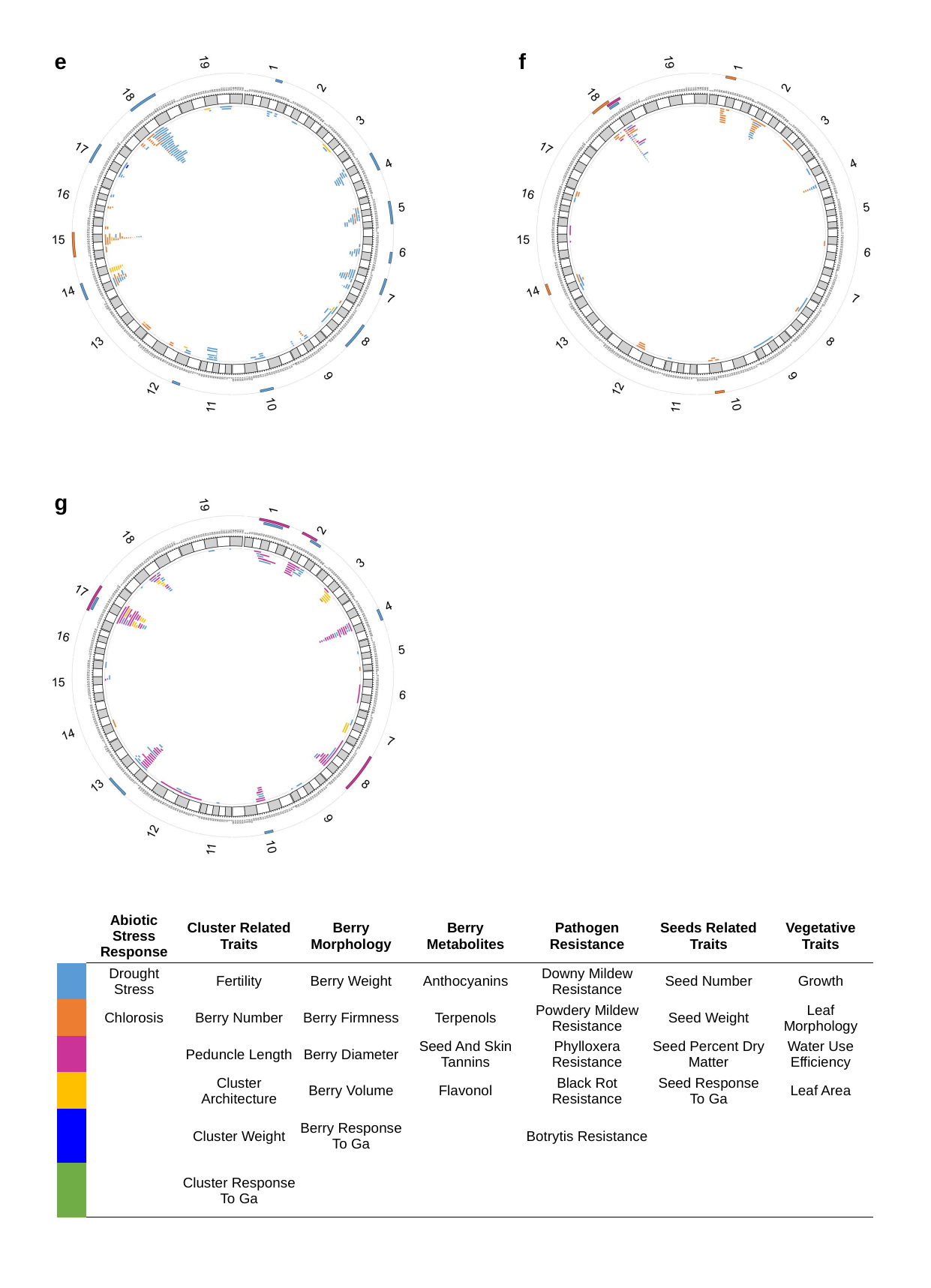

e
f
g
| | Abiotic Stress Response | Cluster Related Traits | Berry Morphology | Berry Metabolites | Pathogen Resistance | Seeds Related Traits | Vegetative Traits |
| --- | --- | --- | --- | --- | --- | --- | --- |
| | Drought Stress | Fertility | Berry Weight | Anthocyanins | Downy Mildew Resistance | Seed Number | Growth |
| | Chlorosis | Berry Number | Berry Firmness | Terpenols | Powdery Mildew Resistance | Seed Weight | Leaf Morphology |
| | | Peduncle Length | Berry Diameter | Seed And Skin Tannins | Phylloxera Resistance | Seed Percent Dry Matter | Water Use Efficiency |
| | | Cluster Architecture | Berry Volume | Flavonol | Black Rot Resistance | Seed Response To Ga | Leaf Area |
| | | Cluster Weight | Berry Response To Ga | | Botrytis Resistance | | |
| | | Cluster Response To Ga | | | | | |
